# Concurrent Presentation of Pustular Psoriasis and Late-Onset Rheumatoid Arthritis: A Diagnostic Challenge in a Community Hospital Setting

**DOI:** 10.7759/cureus.50278

**Published:** 2023-12-10

**Authors:** Ryoka Chayama, Shiho Amano, Satoshi Ofuji, Chiaki Sano, Ryuichi Ohta

**Affiliations:** 1 Family Medicine, Shimane Prefectural Central Hospital, Izumo, JPN; 2 Community Care, Unnan City Hospital, Unnan, JPN; 3 Dermatology, Unnan City Hospital, Unnan, JPN; 4 Community Medicine Management, Shimane University Faculty of Medicine, Izumo, JPN

**Keywords:** family medicine, general medicine, combination therapy management, elderly-onset rheumatoid arthritis, overlapping symptoms, diagnostic challenge, autoimmune disorders, polyarticular pain, rheumatoid arthritis, pustular psoriasis

## Abstract

The coexistence of autoimmune diseases presents a significant diagnostic challenge in clinical practice, particularly in settings with limited resources. This case report details a rare instance of pustular psoriasis occurring concurrently with rheumatoid arthritis, underscoring the complexities involved in diagnosing overlapping autoimmune disorders. A 70-year-old male with a history of chronic heart failure, atrial fibrillation, and other comorbidities presented to a rural community hospital with a year-long persistent rash and joint and back pain. Physical examination and blood tests revealed high inflammatory markers. A dermatological assessment, including a skin biopsy, diagnosed generalized pustular psoriasis. However, the atypical presentation of acute polyarthritis led to further investigations, revealing elevated rheumatoid factor and anti-citrullinated protein antibody levels, resulting in a diagnosis of late-onset rheumatoid arthritis. The patient underwent a comprehensive treatment regime, including prednisolone, cefazolin, oral terbinafine, methotrexate, and infliximab, leading to gradual symptom improvement to the previous activity of daily life and discharge on the 27th day of hospitalization. This case illustrates the diagnostic intricacies in identifying concurrent autoimmune disorders and highlights the crucial role of general physicians in systematically approaching complex cases in resource-limited settings. It emphasizes the need for heightened clinical vigilance and a multifaceted diagnostic approach when managing patients with overlapping rheumatic symptoms, advocating for consideration of coexisting conditions in autoimmune diseases.

## Introduction

Pustular psoriasis is a significant dermatological condition that frequently manifests systemic effects, particularly musculoskeletal symptoms [1]. The clinical challenge becomes particularly complex when this condition coexists with other rheumatic disorders, a rarity that introduces additional diagnostic and therapeutic difficulties [2]. There is little research showing the coexistence of pustular psoriasis and rheumatoid arthritis.

In this paper, we present a noteworthy case of an elderly man experiencing an acute exacerbation of pustular psoriasis, accompanied by severe polyarticular pain. Detailed diagnostic evaluations revealed an unexpected concurrent diagnosis of rheumatoid arthritis. This dual diagnosis is uncommon and presents unique challenges [3]. The overlapping symptoms and potential contradictions in treatment for these conditions can perplex even experienced clinicians [4].

This case report highlights the diagnostic complexities in identifying the coexistence of rheumatoid arthritis and pustular psoriasis. This case report emphasizes the importance of increased clinical awareness and a comprehensive approach to managing overlapping rheumatic conditions in general practice [5]. Through sharing our findings, we aim to enhance understanding and provide practical strategies for effectively managing such intricate clinical situations in future medical practice.

## Case presentation

A 70-year-old male patient presented to the outpatient department of a rural community hospital with a year-long persistent rash on his back, joint pain, and back pain. Ten days before admission, he experienced increased pain in both shoulders and the left elbow. An orthopedic clinic had previously suggested a rotator cuff injury in his right shoulder. Seven days before admission, a generalized rash developed, worsening over the following days. His medical history was significant for chronic heart failure, atrial fibrillation, alcohol dependency, gout, and hypertension, managed with allopurinol of 100mg daily, carvedilol of 10 mg daily, apixaban of 10 mg daily, furosemide of 10 mg daily, and enalapril of 5 mg daily.

**Figure 1:**
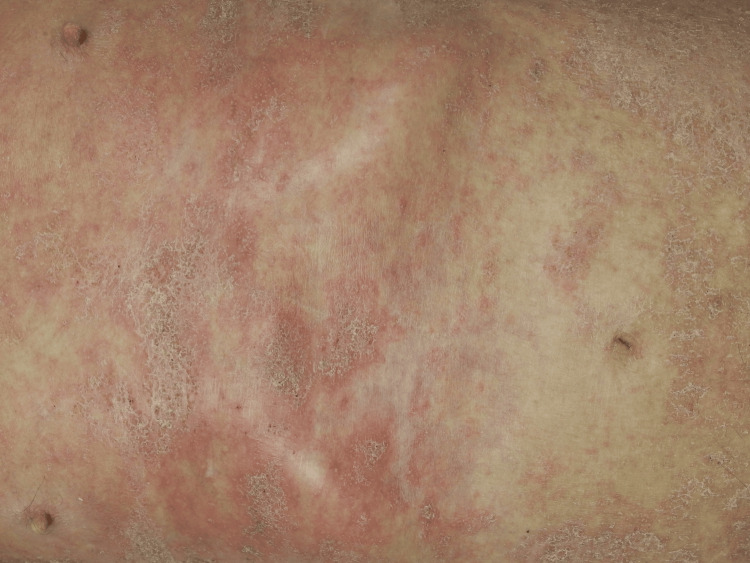
Irregular erythema with crusts on the trunk

Upon arrival, his vital signs were stable: blood pressure 145/89 mmHg, pulse rate 120 beats/min, respiratory rate 21 breaths/min, body temperature 37.2°C, and oxygen saturation 98% on room air. He was alert and oriented. Physical examination revealed bilateral joint warmth, swelling, and tenderness in the shoulders, elbow, wrist, knee, and ankle joints. Tenderness was noted at the biceps brachii attachment. Skin examination showed desquamation on limb extensor surfaces and auricles, irregular erythema with crusts on the trunk, and scattered small pustules with erythema on lower limbs (Figure 1).

No chest, abdominal, or neurological abnormalities were observed. Blood tests showed high inflammatory markers of the rate of neutrophil, erythrocyte sedimentation rate, and C-reactive protein (Table 1).

**Table 1:** Initial laboratory data of the patient Na, sodium; K, potassium; Cl, chloride; CRP, C-reactive protein; Ig, immunoglobulin

Parameter	Level	Reference
White blood cells	9.90	3.5–9.1 × 10^3^/μL
Neutrophils	78.6	44.0–72.0%
Lymphocytes	7.6	18.0–59.0%
Hemoglobin	12.1	11.3–15.2 g/dL
Hematocrit	35.5	33.4–44.9%
Mean corpuscular volume	103.7	79.0–100.0 fl
Platelets	22.8	13.0–36.9 × 10^4^/μL
Erythrocyte sedimentation rate	79	2–10 mm/hour
Total protein	6.7	6.5–8.3 g/dL
Albumin	3.0	3.8–5.3 g/dL
Total bilirubin	0.8	0.2–1.2 mg/dL
Aspartate aminotransferase	20	8–38 IU/L
Alanine aminotransferase	13	4–43 IU/L
Lactate dehydrogenase	244	121–245 U/L
Blood urea nitrogen	22.6	8–20 mg/dL
Creatinine	0.77	0.40–1.10 mg/dL
Serum Na	136	135–150 mEq/L
Serum K	4.1	3.5–5.3 mEq/L
Serum Cl	100	98–110 mEq/L
Ferritin	223.6	14.4–303.7 ng/mL
CRP	16.62	<0.30 mg/dL
IgG	1862	870–1700 mg/dL
IgM	101	35–220 mg/dL
IgA	497	110–410 mg/dL

**Figure 2:**
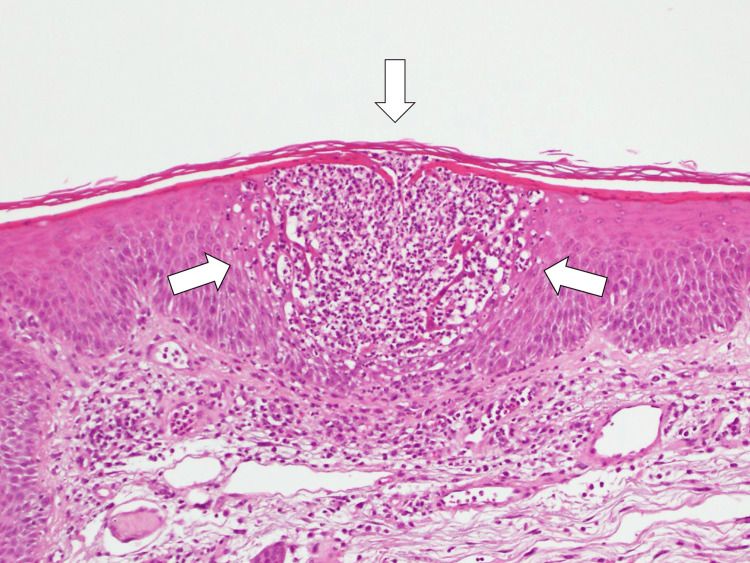
Histopathological photomicrograph of the skin revealing intraepidermal pustules filled with neutrophils in the superficial layer of the skin (white arrows) (hematoxylin and eosin staining ×100)

A dermatology consultation resulted in a skin biopsy, which, combined with the clinical presentation of painful generalized erythema and multiple pustules, led to a diagnosis of generalized pustular psoriasis accompanied by typical pathological findings and concomitant cellulitis (Figure 2).

The severity and atypical nature of the polyarticular pain and acute polyarthritis suggested a different etiological basis for arthritis. Elevated rheumatoid factor (500 IU/ml, reference <15 IU/ml) and anti-citrullinated protein antibody (chemiluminescent enzyme immunoassay) (95.7 U/ml, reference <5 U/ml) levels, alongside a negative antinuclear antibodies test, pointed to concurrent diagnosis of late-onset rheumatoid arthritis based on American College of Rheumatology/European League Against Rheumatism 2010 score of seven.

The patient's treatment regimen included prednisolone (0.5 mg/kg) per oral for one week, addressing both pustular psoriasis and psoriatic arthritis. Cefazolin (4 g/day) was administered for five days to treat a suspected secondary infection. Oral terbinafine was prescribed for tinea corporis. Despite initial treatment, symptoms of elderly-onset rheumatoid arthritis persisted, and the pustular psoriasis did not improve sufficiently. On the seventh day of hospitalization, methotrexate (6 mg/week) and infliximab (300 mg) biweekly infusions were added. This treatment led to gradual symptom alleviation and a trend toward resolution of the generalized pain and rash, especially the disappearance of crusts and erythema on the skin. The patient showed significant improvement in rehabilitation and was discharged on the 27th day of hospitalization.

## Discussion

In the dynamic realm of clinical medicine, the concurrent presentation of multiple diseases poses unique diagnostic challenges, as exemplified by our case of pustular psoriasis coinciding with rheumatoid arthritis. Pustular psoriasis, primarily known for its distinctive skin manifestations such as white pustules, red and tender skin, and peeling and scaling, becomes increasingly complex when systemic symptoms overlap with other rheumatic conditions, potentially mimicking or obscuring them [6]. This complexity is particularly pronounced in community hospitals, where resources and specialist availability may be limited, placing general physicians at the forefront of these diagnostic challenges [7]. Our case emphasizes the necessity for a refined and systematic approach, highlighting the pivotal role of general physicians in identifying the interplay between autoimmune disorders and their potential overlap with other rheumatic diseases [8].

This case illustrates how the systemic symptoms of pustular psoriasis can create intricate and potentially misleading clinical scenarios [9]. The symptomatic overlap with other rheumatic diseases, such as rheumatoid arthritis, demands careful evaluation and a comprehensive investigative strategy [10]. In this case, initially, systemic inflammation of pustular psoriasis potentially caused polyarticular pain. However, the prevalence of the involved inflamed joints was not typical for psoriasis, leading to further investigation of rheumatoid arthritis. In community hospitals, where general physicians often handle diverse medical cases, a systematic approach to diagnosis is crucial, considering common clinical courses of diseases [11,12]. Recognizing the potential coexistence of diseases, particularly in autoimmune disorders, is vital for improving patient outcomes.

When managing multiple symptoms, a comprehensive perspective on autoimmune disorders is imperative. In our case, the diagnosis of rheumatoid arthritis could be possible from a comprehensive perspective of clinical reasoning. In this case, pustular psoriasis can be caused by the abnormality of helper T cells, sharing with the pathophysiology of rheumatoid arthritis and other autoimmune disease [13]. The landscape of autoimmune diseases is vast and interconnected [13], and patients presenting with one autoimmune condition may have underlying or concurrent rheumatic conditions [14]. General physicians should maintain high suspicion and consider a wide range of differential diagnoses, exploring the possibility of coexisting conditions [15].

General physicians' approach should encompass multiple clinical perspectives when dealing with autoimmune diseases [16,17]. While specialist input is invaluable, general physicians, with their broad understanding of medicine, are uniquely placed to identify overlaps and connections in disease presentations. In this case, general physicians can treat the coexistence of pustular psoriasis and rheumatoid arthritis with prednisolone and infliximab based on the understanding of the pathophysiology of these diseases [18]. By acknowledging the diverse development pathways of rheumatic diseases within autoimmune contexts, general physicians can bridge diagnostic gaps and provide comprehensive patient care.

## Conclusions

This case report underscores the diagnostic complexities in identifying overlapping autoimmune disorders, specifically the co-presentation of pustular psoriasis and rheumatoid arthritis. It highlights the critical role of general physicians in systematically approaching such multifaceted cases, particularly in community hospital settings where resources may be limited. For the effective treatment of the coexistence of pustular psoriasis and rheumatoid arthritis, awareness and a comprehensive diagnostic strategy about the pathophysiology and similarity of rheumatic diseases are essential, leading to effective management of coexisting conditions in autoimmune diseases in rural contexts.
